# On migration of primary/peritectic interface during interrupted directional solidification of Sn-Ni peritectic alloy

**DOI:** 10.1038/srep24512

**Published:** 2016-04-14

**Authors:** Peng Peng, Xinzhong Li, Jiangong Li, Yanqing Su, Jingjie Guo, Hengzhi Fu

**Affiliations:** 1 Institute of Materials Science and Engineering, Lanzhou University, Lanzhou 730000, China; 2 MOE Key Laboratory for Magnetism and Magnetic Materials, Lanzhou University, Lanzhou 730000, China; 3 School of Materials Science and Engineering, Harbin Institute of Technology, Harbin 150001, PR China

## Abstract

The migration of the primary/peritectic interface in local isothermal condition is observed in dendritic structure of Sn–Ni peritectic alloy after experiencing interrupted directional solidification. It was observed that this migration of primary Ni_3_Sn_2_/peritectic Ni_3_Sn_4_ interface towards the primary Ni_3_Sn_2_ phase was accompanied by migration of liquid film located at this interface. The migration velocity of this interface was confirmed to be much faster than that of peritectic transformation, so this migration was mostly caused by superheating of primary Ni_3_Sn_2_ phase below T_P_, leading to nucleation and migration of liquid film at this interface. This migration can be classified as a kind of liquid film migration (LFM), and the migration velocity at the horizontal direction has been confirmed to be much faster than that along the direction of temperature gradient. Analytical prediction has shown that the migration of liquid film could be divided into two stages depending on whether primary phase exists below T_P_. If the isothermal annealing time is not long enough, both the liquid film and the primary/peritectic interface migrate towards the primary phase until the superheated primary phase has all been dissolved. Then, this migration process towards higher temperature is controlled by temperature gradient zone melting (TGZM).

Three different phases are involved during peritectic solidificaiton^1^: primary α phase, peritectic β phase and the liquid phase L. Peritectic solidification is composed of three different stages^1^: peritectic reaction L + α → β, subsequent liquid precipitation L → β and peritectic transformation α → β. After peritectic reaction, the peritectic β phase which is produced by peritectic reaction can separate primary α phase and liquid phase. Further growth of β phase by solid state diffusion is accompanied by simultaneous disappearance of α phase, and this stage is termed as peritectic transformation. Besides peritectic transformation, the peritectic β phase can also be foremd through precipitation from the liquid phase L. The driving force of this solid-state transformation is the diffusion driven by the compositional difference 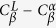 across β phase. Here 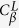 and 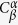 are the concentrations of peritectic β phase in equilibrium with the liquid and primary α phase, respectively^1^,^2^,^3^,^4^. Researches in isothermal condition^5^,^6^ or slow cooling solidification^2^,^4^,^7^,^8^,^9^, especially in Fe-C^4^,^7^,^8^ and Fe-Ni^9^ peritectic alloys have shown that peritectic transformation could not be simply ignored. So interrupted directional solidification has been used to analyze peritectic transformation in many alloy systems. During interrupted directional solidification, local isothermal condition is realized through a stationary standing of the samples after downward directional solidification has been carried out. The feature of peritectic transformation is usually assumed to be the migration of primary/peritectic interface towards the inner part of primary phase^10^,^11^,^12^ in isothermal condition. Although investigation on peritectic transformation can be realized through analyzing the migration of primary/peritectic interface during interrupted directional solidification, in fact, this migration process of primary/peritectic interface is not solely influenced by peritectic transformation. During directional solidification, when the solid phase has been superheated above the solidus temperature, independent melt nuclei ahead of the melting front can also be observed besides melting which starts at grain boundaries^13^.

Nucleation of liquid films in the interior of the solid grains has also been found in directional melting experiments which have been carried out in some alloys^14^,^15^,^16^. During the preparation of initial conditions in directional solidification, nucleation of liquid films in the solid phase and migration of them can lead to microstructure evolution in the mushy zone between the complete solid zone and the complete liquid zone^17^. Migration of liquid films in the mushy zone is controlled by different mechanisms in the mushy zone. Due to the temperature gradient zone melting (TGZM) effect by temperature gradient, migration of liquid films into the complete liquid zone can be observed^18^,^19^. Besides, migration of a thin liquid in a mostly solid structure can also be attributed to the liquid film migration (LFM)^18^,^20^, which is one of a number of chemically induced grain boundary migration phenomena. It occurs when a liquid film between two adjacent grains migrates laterally under the influence of a diffusion gradient^20^,^21^,^22^,^23^,^24^,^25^,^26^,^27^,^28^,^29^. Simultaneous melting and solidification are involved in the mushy zone during these processes, which might lead to position change of the interfaces between different phases^18^. From the experiments and the corresponding theoretical interpretation on nucleation and migration of the liquid films in a temperature gradient, it therefore can be concluded that the influences of these effects on the migration of liquid films and corresponding microstructural evolution should not be simply neglected.

In peritectic systems, the nucleation and migration of the liquid films can lead to the change in both the position and morphology of the primary/peritectic interface where the liquid films located. In addition to the solid/liquid interface, the primary/peritectic interface is formed after peritectic reaction like grain boundaries in single phase alloys. Although it has been theoretically predicted that the nucleation and migration of liquid films could take place at primary/peritectic interface^18^, it has not been experimentally confirmed yet. And query still exists in how the migration of liquid film can be effective in migration of primary/peritectic interface together with peritectic transformation. The aim of this study is to shed light on the stability and variation of primary/peritectic interface of a peritectic system in local isothermal condition. For this reason, the Sn-Ni peritectic system in which both the primary Ni_3_Sn_2_ phase and peritectic Ni_3_Sn_4_ phase are intermetallic compounds with narrow solubility ranges has been selected. In this kind of peritectic system, the driving force for peritectic transformation 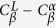 is quite limited, helping to reduce the influence of peritectic transformation on solidification microstructure. To investigate and characterize the position and morphology of primary/peritectic interface in local isothermal condition, the interrupted directional solidification in which the isothermal condition is realized through a stationary standing of the samples after downward directional solidification has been carried out. Then, based on the experimental observations on the microstructure, especially the variation in both position and morphology of the primary/peritectic interface, analytical models have been established to describe the migration process of liquid film and primary/peritectic interface in isothermal condition in this peritectic system.

## Results

**Figure 1:**
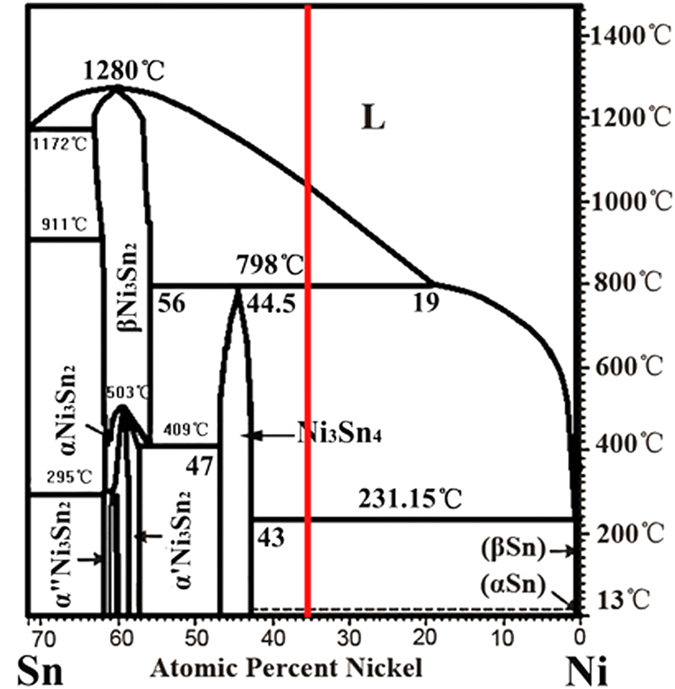
Relevant part of Sn-Ni binary phase diagram^30^.

**Figure 2:**
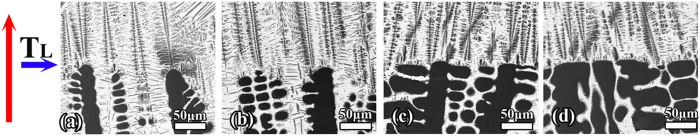
Microstructure of the solid/liquid interface of the longitudinal sections of directionally solidified Sn–36at%Ni alloy after different isothermal annealing time t: (**a**) 120 s; (**b**) 300 s; (**c**) 900 s; (**d**) 1800 s.

**Figure 3:**
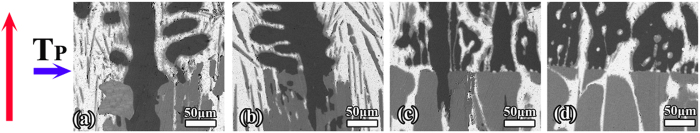
Microstructure of the peritectic interface of the longitudinal sections of directionally solidified Sn–36at%Ni alloy after different isothermal annealing time t: (**a**) 120 s; (**b**) 300 s; (**c**) 900 s; (**d**) 1800 s.

It can be observed from Fig. 1 that under equilibrium condition^30^ solidification of Sn–36at.%Ni alloy begins at T_L_ = 1040 °C with a primary precipitation of Ni_3_Sn_2_ phase: L → Ni_3_Sn_2_; followed by a peritectic reaction at T_P_ = 798 °C: L + Ni_3_Sn_2_ → Ni_3_Sn_4_; then at T_E_ = 231.15 °C, the remaining liquid was quenched as the eutectic (Ni_3_Sn_4_ + Sn). In this work, Ni_3_Sn_2_/Ni_3_Sn_4_ phases are the primary α/β phases in the Sn-Ni peritectic system. In the following analysis and discussions, “Ni_3_Sn_2_” and “Ni_3_Sn_4_” are used when illustrating the experimental observations in this work while “α” and “β” are used when discussing a general solidification case. The Typical BSE (backscattered electron) microstructures of the longitudinal sections of directionally solidified Sn–36at%Ni alloy after different isothermal annealing time are shown in Figs 2 and 3. The vertical red arrows show the growth direction of the samples, the positions of the solid/liquid interface and the peritectic interface are indicated by the small blue arrows. The dark/grey phases represent primary Ni_3_Sn_2_/peritectic Ni_3_Sn_4_ phases, respectively; and the remaining white is the eutectic^4^,^8^,^9^. It is worth noting that four different zones can be observed in the samples after isothermal annealing: the complete liquid zone, the (Ni_3_Sn_2_ + liquid) mushy zone, the (Ni_3_Sn_2_ + Ni_3_Sn_4_ + liquid) mushy zone, below T_E_ is the complete solid region. During isothermal annealing, the (Ni_3_Sn_2_ + Ni_3_Sn_4_ + liquid) mushy zone gradually changes into the (Ni_3_Sn_4_ + liquid) one.

More importantly, the position and morphology of the primary/peritectic interface changes in several aspects during isothermal annealing. The most obvious one is that, the volume fraction of primary Ni_3_Sn_2_/peritectic Ni_3_Sn_4_ phase gradually decreases/increases with increasing isothermal annealing time t. Simultaneous migration of the primary/peritectic interface towards the primary Ni_3_Sn_2_ phase can be observed. Besides, distinct from the structure feature of peritectic transformation^1^, the volume fraction of peritectic Ni_3_Sn_4_ phase is larger at higher temperature below T_P_ . This means that the Ni_3_Sn_2_/Ni_3_Sn_4_ interface migrates more quickly at higher temperatures towards primary Ni_3_Sn_2_ phase. In addition, a number of liquid films which are elongated along temperature gradient can be observed in solid phases after isothermal annealing. Most of these elongated liquid films are located at the Ni_3_Sn_2_/Ni_3_Sn_4_ interface. Finally, after isothermal annealing, the relatively flat Ni_3_Sn_2_/Ni_3_Sn_4_ interface becomes much more curved, and the distances from this curved Ni_3_Sn_2_/Ni_3_Sn_4_ interface to the center of primary dendrite stem are different at different local temperatures.

It is interesting to note that the distribution of liquid films is dependent on the isothermal annealing time t. When the isothermal annealing time t is above 15 min, the (Ni_3_Sn_2_ + Ni_3_Sn_4_ + liquid) mushy zone becomes the (Ni_3_Sn_4_ + liquid) mushy zone. In this case, the Ni_3_Sn_2_/Ni_3_Sn_4_ interface disappears in the (Ni_3_Sn_4_ + liquid) mushy zone. Contrary to the regular arrangement of solid phases in the (Ni_3_Sn_4_ + liquid) mushy zone, the arrangement of Ni_3_Sn_2_ phase in the (Ni_3_Sn_2_ + liquid) mushy zone is quite irregular and complex. The Ni_3_Sn_2_ grains with irregular morphology exist at the bottom of the (Ni_3_Sn_2_ + liquid) mushy zone, and numerous liquid droplets of different sizes and morphologies are located inside these grains. In addition, this irregularity of both Ni_3_Sn_2_ grains and the liquid droplets within them extends from the bottom to the top of (Ni_3_Sn_2_ + liquid) mushy zone with increasing isothermal annealing time.

## Discussion

As has been proposed by St John and Hogan^2^ and Tichener and Spittle^5^, the rapid formation of β phase through peritectic transformation will occur easily if the following two requirements can be satisfied: the composition gaps between α and β phases and β and liquid phases are small; the composition range of peritectic β phase is large. It is in general believed that the migration of primary/peritectic interface can only be caused by peritectic transformation after peritectic reaction. Complete peritectic transformation which is due to fast rate of peritectic transformation has already been observed in directionally solidified Fe-C^6^ and Fe-5.01at.%Ni alloys^9^. However, since the diffusion coefficient in a liquid phase is larger than that in a solid phase by 3–4 orders of magnitude, the rate of peritectic transformation is generally extremely slow in most of other alloy systems. For Sn-36at.%Ni alloy, its solute concentration is much larger than these Fe-C and Fe-Ni peritectic alloys of dilute concentrations, and the diffusion coefficient of C in Fe is much faster than that of Ni in Sn. In addition, it can be found from Fig. 1 that the driving force of peritectic transformation, namely, the compositional difference 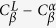 across peritectic β phase is quite small in Sn-Ni peritectic alloys due to the narrow range of compositions of peritectic Ni_3_Sn_4_ phase^31^. So, the migration distance of primary/peritectic interface due to peritectic transformation should not be fast during isothermal annealing in this work.

**Figure 4:**
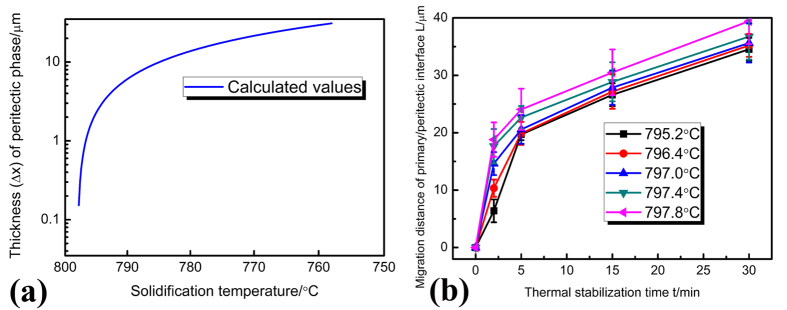
Determination of whether peritectic transformation plays an important role in the migration process: (**a**) the calculation results on the variation of the thickness *∆x* by peritectic transformation during isothermal annealing; (**b**) experimental measurement of the migration distance of the primary/peritectic interface L after isothermal annealing.

Thus, whether peritectic transformation plays a major role in thickening (growth) of peritectic Ni_3_Sn_4_ phase during isothermal annealing should be identified first. The description of peritectic transformation has been presented by many researches^2^,^5^,^10^,^12^. Here, the analytical presentation by John *et al*.^2^ is used:


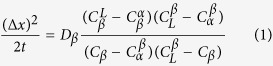


where ∆*x* is the thickness of peritectic phase formed through peritectic transformation. The explanations of these concentrations are as follows: 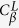 and 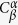 are the concentrations of peritectic β phase in equilibrium with the liquid and primary α phase, respectively^1^,^2^,^3^,^4^; 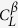 and 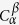 are the concentrations of liquid phase/primary α phase in equilibrium with peritectic β phase, respectively^1^,^2^,^3^,^4^; *C*_*β*_ is average concentration of the peritectic β phase. *D*_*β*_ is the average diffusion coefficient of peritectic β phase, and can be expressed as:


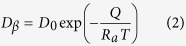


where *D*_0_ is a physical constant depending on alloy composition, *R*_*a*_ is gas constant, 8.314 J/mol·K. *Q* is the activation enthalpy, respectively. In the Sn–Ni peritectic system, the physical constant *D*_0_  = 5 × 10^−9^m^2^/s, the activation enthalpy *Q* = 19150J/mol is obtained by averaging the values from previous researches^32^,^33^. The calculation result obtained through equation (1) and (2) is illustrated in Fig. 4a. The dependences of the migration distance of primary/peritectic interface L on isothermal annealing temperature and isothermal annealing time are shown in Fig. 4b. This distance L equals to the increase in thickness of peritectic Ni_3_Sn_4_ phase during isothermal annealing. L increases more quickly at higher temperatures, which means that the Ni_3_Sn_2_/Ni_3_Sn_4_ interface migrates more quickly towards the Ni_3_Sn_2_ phase at higher temperatures. Comparison between Fig. 4a,b shows that the peritectic phase formed by peritectic transformation only accounts for a small proportion of the total migration distance of the Ni_3_Sn_2_/Ni_3_Sn_4_ interface. Besides, it can be found from Fig. 4a that the growth of peritectic Ni_3_Sn_4_ phase by peritectic transformation is more obvious at lower temperatures. This is different from the dependence of L on temperature (Fig. 4b), which indicates that the migration distance L is larger at higher temperatures. Although the contribution of peritectic transformation to the migration of the α/β interface can not be neglected, its influence can not be overestimated either. It is therefore concluded that there exists other mechanisms which are dominant during the migration of α/β interface.

During interrupted directional solidification, the solid phases in the mushy zone which is defined from T_E_ to T_L_ are superheated due to the temperature gradient imposed on the stationary sample. Nucleation of liquid phase occurs in solid phases of the mushy zone since the existence of superheated solid where the coexistence of liquid and solid is possible^16^. Although liquid phase can be nucleated in the interior of solid phases, melting is recognized as a surface-initiated process^34^. Melting begins as a continuous vibrational instability, and nucleation of liquid phase is inclined to occur at the surface of a solid-solid boundary. Therefore, melting is assumed to always start at a grain boundary in single phase alloys^35^. In peritectic systems, nucleation of liquid phase is more complex. The solid phases annealed at temperatures above T_E_ are presumed to remelt, while those below T_E_ are assumed to remain solid. As shown in Fig. 1a, α/β interface is not stable in peritectic systems below T_P_ while stable existence of the β/L interface is definitive. As a result, liquid phase is most likely to nucleate at α/β interface during isothermal annealing. The experimental results shown in Fig. 3 confirm the nucleation of liquid phase at Ni_3_Sn_2_/Ni_3_Sn_4_ interface.

When liquid phase is nucleated at α/β interface, the solute concentration of the liquid film should be in local equilibrium with its neighboring solid phases. Thus, the solute concentration is not uniform within the liquid film since the liquid film is in equilibrium with both primary and peritectic phases. As a result, a concentration gradient is produced within the liquid film. Since the Sn concentration of liquid phase which is in contact with peritectic Ni_3_Sn_4_ phase is lager than that in contact with primary Ni_3_Sn_2_ phase, mass transport from the Ni_3_Sn_4_/liquid interface to the Ni_3_Sn_2_/liquid interface occurs. In this case, melting/solidification occur at the Ni_3_Sn_2_/liquid and Ni_3_Sn_4_/liquid interfaces to keep local equilibrium. And both the liquid film and the Ni_3_Sn_2_/Ni_3_Sn_4_ interface migrate towards primary Ni_3_Sn_2_ phase as long as this melting/solidification process maintains.

**Figure 5:**
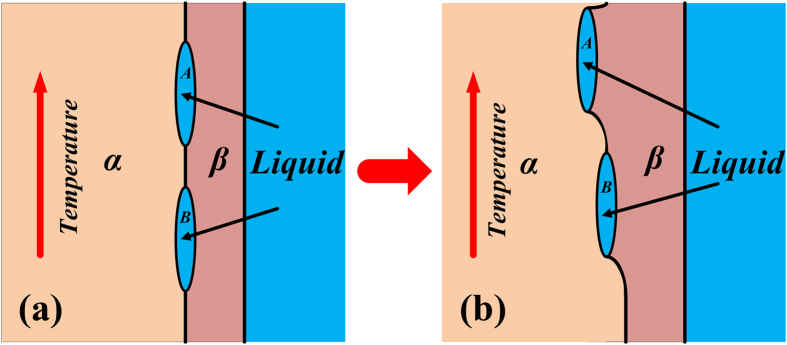
Illustration of the migration process of liquid film within primary dendrite arm.

**Figure 6:**
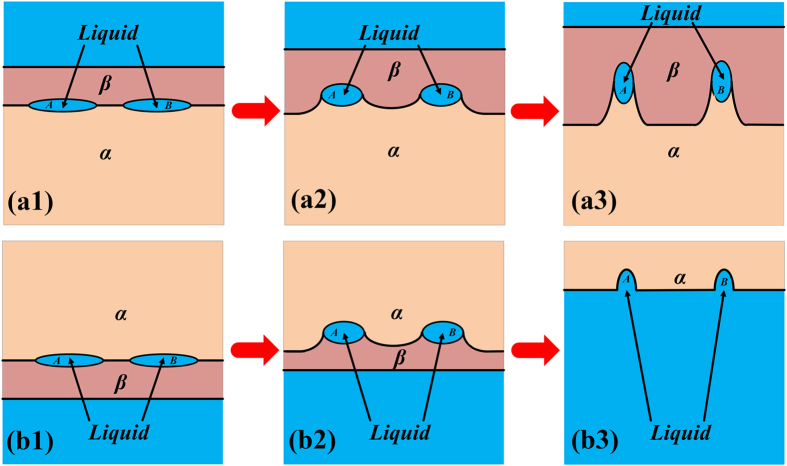
Illustration of the migration process of liquid film within secondary dendrite arm.

The migration process of liquid films shown in Fig. 2 can be divided into different types in this work in terms of the locations where these films exist during isothermal annealing. They are: migration of liquid film within primary dendrite arm and that within secondary arm. These two kinds of migration are illustrated in Figs 5 and 6, respectively. The analytical models based on these illustrations are proposed to describe this migration process. The establishment of these analytical models has been presented in Supplementary Information. Generally speaking, our experimental observations show that not only the migration direction, but also the morphology of the liquid film and the α/β interface are changing during the migration process.

When primary Ni_3_Sn_2_ phase below T_P_ has been dissolved, liquid films can also be formed in superheated solid phases due to the imposed temperature gradient. As shown in Fig. 3c,d, in this case, nucleation of liquid films at α/β interface is confirmed through the existence of these liquid films in primary Ni_3_Sn_2_ phase at temperature slightly higher than T_P_ . Therefore, lateral migration of liquid films which is perpendicular to temperature gradient can be neglected while migration of liquid films along temperature gradient is dominant. Enlarged view of α/β interface at T_P_ shows that nucleation of liquid films can easily occur at α/β interface at T_P_. In addition, the morphology of Ni_3_Sn_2_ grains is not regular due to the migration of liquid phase. It should be noted that these droplets are gradually elongated during migration, while the amount of them decreases. In addition, the shape of liquid phase changes from spherical to elongated oval. This because that the Sn concentration at the lower temperature of the liquid droplet is higher than that at the higher temperature of the liquid droplet due to the imposed temperature gradient. So the remelting rate at higher temperature of liquid droplet should be larger than the resolidification rate at the lower temperature of liquid droplet. As a result, the droplets can be elongated along temperature gradient.

Disappearance of these liquid films in the (Ni_3_Sn_2_ + liquid) mushy zone can be attributed to irregular morphology of Ni_3_Sn_2_ grains in this mushy zone. Compared with the arrangement of Ni_3_Sn_4_ grains below T_P_ , the arrangement of Ni_3_Sn_2_ grains above T_P_ is far from uniform. Since the liquid films located within these irregular Ni_3_Sn_2_ grains can be easily in contact with the liquid phase outside of these grains, disappearance of them occurs quite easily. Irregular morphology of Ni_3_Sn_2_ grains can be caused by the following two reasons. On the one hand, although the primary dendrite arms are more or less parallel to temperature gradient, remelting/resolidification of secondary and higher order branches during isothermal annealing leads to irregular morphology of the Ni_3_Sn_2_ grains. On the other hand, the volume fraction of liquid is higher at the lower part of (Ni_3_Sn_2_ + liquid) mushy zone due to nucleation of liquid phase at the Ni_3_Sn_2_/Ni_3_Sn_4_ interface at T_P_ . Therefore, thermosolutal convection due to the density difference between Ni and Sn atoms is produced. The morphology of Ni_3_Sn_2_ grains should be more complex in existence of thermosolutal convection. Furthermore, it is interesting to note that the migration of liquid phase influences the non-uniform morphology of Ni_3_Sn_2_ grains which can also lead to the disappearance of liquid droplets themselves.

**Figure 7:**
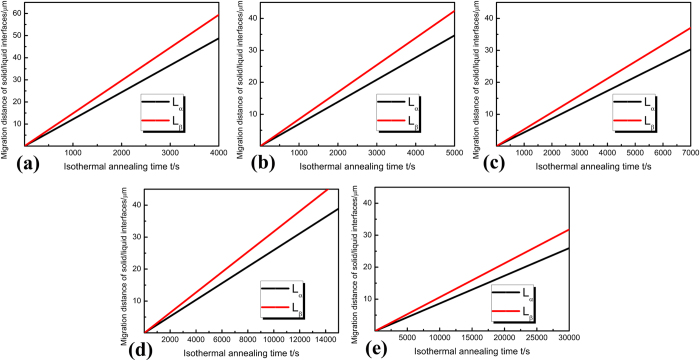
The migration distances of both the α/L and β/L interfaces within primary dendrite arm under different isothermal annealing temperatures: (**a**) 797.8 °C; (**b**) 797.4 °C; (**c**) 797.0 °C; (**d**) 796.4 °C; (**e**) 795.2 °C.

Predictions of the migration distances of both the α/L(Ni_3_Sn_2_/L) and β/L(Ni_3_Sn_4_/L) interfaces have been presented in Fig. 7. The analytical prediction shows that the migration velocities of liquid film and Ni_3_Sn_2_/Ni_3_Sn_4_ interface are faster at higher temperatures, which is consistent with the experimental observation. In this case, the migration distances L_α_ and L_β_ are all larger at higher temperatures. It is interesting to note that since the resolidification velocity at the tail of liquid film (β/L interface) is larger than the remelting velocity at the front of liquid film (α/L interface), so the thickness of the liquid film in the direction perpendicular to temperature gradient *l* should decrease as migration proceeds. Once the difference, L_β_−L_α_ is shorter than the thickness of the liquid film in the direction perpendicular to temperature gradient *l*, this liquid film is disappeared. Although liquid films gradually disappear during the migration process, nucleation of liquid films at the Ni_3_Sn_2_/Ni_3_Sn_4_ interface continues as superheated solid phases still exist. As a result, migration of liquid films and the Ni_3_Sn_2_/Ni_3_Sn_4_ interface where these films located proceeds till the final dissolution of α phase.

The prediction in Fig. 7 also shows that the duration of a liquid film in primary dendrite arm is shorter at higher temperatures, and is about 8 min in average. This observation verifies the continuous nucleation and migration of liquid films in superheated solid phases. In addition, liquid films having different distances from the center of primary dendrite stem of primary Ni_3_Sn_2_ phase can be observed, which also demonstrates that the nucleation of liquid films is not simultaneous. Furthermore, it can be seen from Fig. 7a that both the remelting (α/L interface) and resolidification (β/L interface) velocities are nearly constant during the migration of liquid film. Strictly speaking, both these velocities are dependent on local temperatures, thus local melt concentrations. So, the liquid films also migrates towards higher temperatures during the migration process, leading to increase of the Ni concentration within the liquid film. However, the migration (remelting/resolidification) velocities of the liquid film which are perpendicular to temperature gradient are much larger than those parallel to temperature gradient, so this variation in melt concentration within the liquid film can be neglected. Therefore, both the remelting and resolidification velocities can be assumed to be constant.

**Figure 8:**
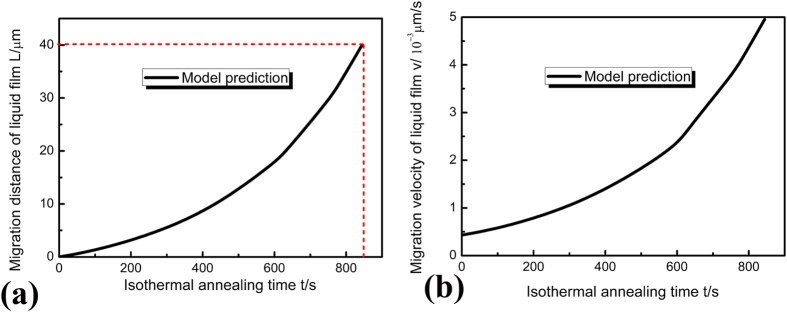
The predictions of the migration of liquid film within the secondary dendrite arm: (**a**) dependence of the migration distance of liquid film L on isothermal annealing time; (**b**) dependence of the migration velocity of liquid film v on isothermal annealing time.

The time a liquid film needs to migrate through a secondary dendrite arm in direction of temperature gradient can also be predicted through the analytical model. Figure 8a shows that the time a liquid film at the back edge of secondary branch needs to migrate through this secondary branch (average length of 40 μm) is about 840 s. This time can be assumed to be the total time required for remelting of secondary dendrite arms during isothermal annealing. Comparison between the experimental results (Fig. 3c) and our predictions shows that this prediction is consistent with present experimental results (secondary dendrite branch has been disappeared in Fig. 3c). The dependence of the migration velocity of liquid film in secondary dendrite arm on isothermal annealing time is shown in Fig. 8b. Analytical prediction shows that the migration velocity of liquid film increases with increasing isothermal annealing time. This is consistent with the real migration process that the liquid films migrate faster at higher temperatures.

The movement of a thin liquid zone in a mostly solid structure is referred to as LFM^18^. If the local geometry is perfectly symmetric, the liquid layer should be stationary at constant temperature. Only if the geometry symmetry is broken can this liquid layer start to migrate. The broken symmetry can be caused by many reasons, like coherency strains, concentration difference across this liquid layer *et al*.^18^. Many mechanisms have been proposed to lead to a migration of liquid film. The coherency strain in the solid phase on one side of the film is assumed to be the driving force for LFM^20^,^21^,^22^,^23^,^24^,^25^,^26^,^27^,^28^,^29^ in several works. The different coherency strains in two adjacent grains are caused by the composition difference across the grain boundary. However, the chemical driving forces should be stronger as compared with coherency strains or interfacial pressure at temperatures close to the melting temperature^18^. In the present work, although the migration of liquid film is similar to that by coherency strains, it is certainly driven by the reduction of superheating. The possible way how superheating could be reduced effectively is a melting/resolidification mechanism. Two different mechanisms for the lateral growth and vertical migration of liquid films were distinguished. Lateral growth of the liquid films is due to superheating of solid whereas the vertical migration is suggested to follow a temperature gradient zone melting (TGZM)^23^ mechanism. The migration of solute rich liquid films inside the mushy zone due to TGZM has also been observed in many alloys^18^. The difference in solute concentration across the liquid film gives rise to a concentration gradient and leads to solute transport during the migration process of liquid film in this work. Diffusion of solute atoms away from a solid/liquid interface causes solidification, whereas solute diffusion towards the interface causes melting. Both interfaces will thus start to migrate in the same direction. At the solidifying interface, solid with equilibrium concentration, i.e. a concentration lower than the former supersaturated concentration, will solidify. Migration of the liquid film through primary Ni_3_Sn_2_ phase leads to a solute concentration change in the solid. Since the primary Ni_3_Sn_2_ phases above T_E_ are all superheated, migration of the liquid film, thus migration of the primary/peritectic interface will continue until the complete dissolution of the superheated primary Ni_3_Sn_2_ phases.

## Conclusion

The migration of primary/peritectic interface has been analyzed in a Sn–Ni peritectic alloy through interrupted directional solidification. And the following conclusions can be made.

1. Liquid film has been observed to nucleate at the primary Ni_3_Sn_2_/peritectic Ni_3_Sn_4_ interface due to the superheating of primary phase below T_P_. And the migration of liquid film plays a major role in the migration of primary/peritectic interface as compared with peritectic transformation.
2. The migration of liquid film at both primary and secondary dendrite arms have been investigated through the newly-established analytical models. This migration process can be classified as a kind of liquid film migration (LFM), while the driving force for this process is the concentration gradient across the liquid film.
3. Migration of liquid phase can be divided into two stages depending on the isothermal annealing time. If this time is not long enough, migration of the liquid film continues until the superheated primary phase has all been dissolved. Then, migration of the liquid film is controlled by temperature gradient zone melting (TGZM).

## Experimental Section

Sn-36at.%Ni peritectic alloy was induction melted under an argon atmosphere from pure Ni and Sn (99.9%). As-cast rods of 3 mm in diameter and 110 mm in length were cut from the ingot. The experiments consisting of melting followed by directional solidification were carried out in a Bridgman-type system consisting of a resistance furnace, a water cooled liquid metal bath filled with liquid Ga–In–Sn alloy, which has been described in previous work^31^. Temperature profiles were measured separately using a PtRh30-PtRh6 thermocouple inserted within a fine alumina tube (0.6 mm in inner diameter) down to the center of the samples. During the pulling process, the thermocouple moved downwards with the sample at the same pulling rate. The temperature gradient close to the solid/liquid interface was deduced from the temperature profiles and was approximately 20 K/mm.

Interrupted directional solidification of Sn-36at.%Ni peritectic alloy can be divided into three successive steps. First, the furnace was heated to 1250 °C to melt the as-cast samples alloy during 2 h, and then was held for 30 min to homogenize the melt. Then, the samples were directional solidified at a growth velocity of 5 μm/s in a constant temperature gradient (20 K/mm). When a predetermined growth distance of 30 mm reached, the samples were kept stationary for different isothermal annealing time (2, 5, 10 and 30 min). Then, the samples were quenched into liquid Ga-In-Sn alloy quickly to preserve the microstructure. And the samples were longitudinally sectioned, polished and etched with a solution of 10 g FeCl_3_-20 ml HCl-180 ml H_2_O for further analysis. The microstructure of the longitudinal section of rods was analyzed by scanning electron microscopy (SEM (Quanta-200)). Energy dispersive X-ray spectrometer (EDS) was applied to determine the composition of phases.

## Additional Information

**How to cite this article**: Peng, P. *et al*. On migration of primary/peritectic interface during interrupted directional solidification of Sn-Ni peritectic alloy. *Sci. Rep*. **6**, 24512; doi: 10.1038/srep24512 (2016).

## Supplementary Material

Supplementary Information
