# Supplementary material for: On migration of primary/peritectic interface during interrupted directional solidification of Sn-Ni peritectic alloy
Source: Sci Rep. 2016 Apr 14;6:24512. doi: 10.1038/srep24512 (PMC4830927; doi:10.1038/srep24512)
Supplement: Supplementary Information [file srep24512-s1.doc]

**Analytical model on migration of the liquid film during isothermal annealing**

**Peng Penga,b[[1]](#footnote-2)*, Xinzhong Li^c^, Jiangong Li^a,b^, Yanqing Su^c^, Jingjie Guo^c^, Hengzhi Fu^c^**

*a. Institute of Materials Science and Engineering, Lanzhou University, Lanzhou 730000, China*

*b. MOE Key Laboratory for Magnetism and Magnetic Materials, Lanzhou University, China*

*c. School of Materials Science and Engineering, Harbin Institute of Technology, Harbin, China*

**1. Assumption of the analytical model**

During the interrupted directional solidification, the influence of the TGZM effect should be taken into consideration since a concentration gradient is produced due to the imposed temperature gradient in the liquid phase. Being influenced by the TGZM effect, liquid phase migrates towards the higher temperatures of the solid phases along the direction of the temperature gradient. As cellular or dendritic morphology can usually be observed in solidified microstructures, the normal direction of the α/β interface might be parallel or perpendicular to, or with an intersection angle *θ* with the temperature gradient. Thus, interaction of the migration of liquid phase by local equilibrium and that by the TGZM effect makes the migration of the α/β interface more complicated. To describe this migration process of both the liquid film and the solid-solid interface more accurately, the following discussion is divided into two types depending on the where the liquid films located: the liquid film, thus the α/β interface within primary dendrite arm and that within secondary dendrite arm.

Some assumptions are made as follows to model the migration of liquid film:

(1) Local equilibrium is established very rapidly at the solid/liquid interface.

(2) Nucleation undercooling is assumed to be 0.

(3) The concentration gradient is assumed to be constant and linear.

(4) The distribution of melt concentration which is parallel to the temperature gradient is assumed to follow the constant straight liquidus lines.

(5) The densities of phases involved are assumed to be the same for simplification.

**2. Migration of primary/peritectic interface within primary dendrite arm**

As shown in Fig. 1(a), in this case, the normal directions of the leading and tailing interfaces of the liquid film are perpendicular to temperature gradient. Since the migration of the liquid film is also influenced by the TGZM effect [1, 2], the liquid film, also the α/β interface will not only migrate laterally towards the center of the primary dendrite stem. Instead, the migration direction of the liquid film migrates has an intersection angle *θ* with the temperature gradient (0*<θ<π/2*). For this reason, the migration of liquid film located at the primary dendrite arm will be analyzed in two directions, ie. parallel and perpendicular to the direction of temperature gradient.

**2.1 Parallel to temperature gradient**

In this case, two triple junctions where the α, β and liquid phases coexist are located, and the equilibrium melt concentration here should be equal to *C_LP_*. However, the distribution of solute concentration within liquid film is more complicated in a temperature gradient. In the direction of temperature gradient, as the solute concentration of liquid phase should follow local equilibrium concentration, the solute concentration of liquid phase should also follow the liquidus distribution of the phase diagram. The morphology of the liquid film located at the primary dendrite arm is illustrated in Fig. 1(a). These two triple junctions are located at points A and C, with solute concentration *C_A_* and *C_C_*. The solute concentration of points B and D are assumed to be $C_{B}=C_{l}^{\alpha,T_{0}}$ and $C_{D}=C_{l}^{\beta,T_{0}}$. Therefore, due to the temperature gradient imposed on this liquid film, solute diffusion due to the solute concentration difference along temperature gradient occurs. Since Sn atoms are rejected into liquid during solidification, the following discussion is based on Sn concentration.

Considering the left part of liquid film ranging from T_a_ to T_b_, by referring to Fig. 1(a), it can be obtained that:

$T_{a}=T_{\alpha}+m_{l}^{\alpha}C_{l}^{\alpha,T_{a}}$ (1a)

$T_{b}=T_{\alpha}+m_{l}^{\alpha}C_{l}^{\alpha,T_{b}}$ (1b)

where $m_{l}^{\alpha}$and $m_{l}^{\beta}$are the liquidus slopes of α/β phases; $C_{l}^{\alpha,T_{a}}$and $C_{l}^{\alpha,T_{b}}$are the melt concentrations at T_a_ and T_b_, respectively. Simultaneously, due to assumptions 3 and 4, concentration gradient resulting from temperature gradient is established within the liquid film:

$T_{a}=T_{b}+GL$ (1c)

Combining the above three equations:

$C_{l}^{\alpha,T_{b}}-C_{l}^{\alpha,T_{a}}=-\frac{GL}{m_{l}^{\alpha}}$ (2)

On the left/right hand side of triple junction A, the liquid phase is in contact with primary/peritectic phase. According to the Fick’s First law, at the α/L point of A:

$J_{1}=D\frac{C_{l}^{\alpha,T_{a}}-C_{l}^{\alpha,T_{b}}}{L}$ (3)

The application of a mass balance for a small displacement *dL_α_* of the triple junction results in the following differential equation:

$J_{2}=C_{l}^{\alpha,T_{a}}\left(k_{\alpha}-1\right)\left.\frac{dL_{\alpha}}{dt}\right|_{T_{a}}$ (4)

$J_{1}=J_{2}$ (5)

$\left.\frac{dL_{\alpha}}{dt}\right|_{T_{a}}=-\frac{DG}{m_{l}^{\alpha}C_{l}^{\alpha,T_{a}}\left(k_{\alpha}-1\right)}$ (6a)

Similarly, for the left hand side of point C:

$\left.\frac{dL_{\alpha}}{dt}\right|_{T_{b}}=\frac{DG}{m_{l}^{\alpha}C_{l}^{\alpha,T_{b}}\left(k_{\alpha}-1\right)}$ (6b)

Therefore, at the right of point A and C, it can be obtained that:

$\left.\frac{dL_{\beta}}{dt}\right|_{T_{a}}=-\frac{DG}{m_{l}^{\beta}C_{l}^{\beta,T_{a}}\left(k_{\beta}-1\right)}$ (7a)

$\left.\frac{dL_{\beta}}{dt}\right|_{T_{b}}=\frac{DG}{m_{l}^{\beta}C_{l}^{\beta,T_{b}}\left(k_{\beta}-1\right)}$ (7b)

Comparison between $\left.\frac{dL_{\alpha}}{dt}\right|_{T_{a}}$and $\left.\frac{dL_{\beta}}{dt}\right|_{T_{a}}$and that between $\left.\frac{dL_{\alpha}}{dt}\right|_{T_{b}}$and $\left.\frac{dL_{\beta}}{dt}\right|_{T_{b}}$shows that both the remelting and resolidification rate are larger at the α/L point, indicating that the morphology of the liquid film is not symmetry during isothermal annealing.

**2.2 Perpendicular to temperature gradient**

At point B one has:

$T_{0}=T_{\alpha}+m_{l}^{\alpha}C_{l}^{\alpha,T_{0}}$ (8a)

At point D:

$T_{0}=T_{\beta}+m_{l}^{\beta}C_{l}^{\beta,T_{0}}$ (8b)

where $C_{l}^{\alpha,T_{0}}$and $C_{l}^{\beta,T_{0}}$are the solute concentrations of liquid at T_0_. Combining the above two equations:

$C_{l}^{\beta,T_{0}}-C_{l}^{\alpha,T_{0}}=\frac{1}{m_{l}^{\beta}}\left[\left(m_{l}^{\beta}-m_{l}^{\alpha}\right)\left(C_{LP}-C_{l}^{\alpha,T_{0}}\right)\right]$ (9)

According to the Fick’s First law, at the α/L interface of B:

$J_{1}=D\frac{C_{l}^{\beta,T_{a}}-C_{l}^{\alpha,T_{0}}}{l}$ (10)

The application of a mass balance for a small displacement *dl_α_* of the triple junction results in the following differential equation:

$J_{2}=C_{l}^{\alpha,T_{0}}\left(k_{\alpha}-1\right)\left.\frac{dl_{\alpha}}{dt}\right|_{T_{0}}$ (11)

$J_{1}=J_{2}$ (12)

$\left.\frac{dl_{\alpha}}{dt}\right|_{T_{0}}=\frac{D}{C_{l}^{\alpha,T_{0}}\left(k_{\alpha}-1\right)m_{l}^{\beta}}\left[\left(m_{l}^{\beta}-m_{l}^{\alpha}\right)\left(C_{LP}-C_{l}^{\alpha,T_{0}}\right)\right]$ (13a)

Similarly, at the β/L interface of D:

$\left.\frac{dl_{\beta}}{dt}\right|_{T_{0}}=\frac{D}{C_{l}^{\beta,T_{0}}\left(1-k_{\beta}\right)m_{l}^{\beta}}\left[\left(m_{l}^{\beta}-m_{l}^{\alpha}\right)\left(C_{LP}-C_{l}^{\alpha,T_{0}}\right)\right]$ (13b)

Comparison between $\left.\frac{dl_{\alpha}}{dt}\right|_{T_{0}}$and $\left.\frac{dl_{\beta}}{dt}\right|_{T_{0}}$shows that the remelting rate at the α/L interface of B is smaller than the resolidificaiton rate at the β/L interface of D, indicating that the liquid film gradually disappears during this migration process. Then, nucleation of liquid films proceeds continuously in the superheating solid phases, and this nucleation and migration process of liquid film continues till the complete dissolution of primary phase below T_P_. The directions of the migration velocities at different locations of the liquid film have been illustrated in Fig. 1(b).

Further comparisons between $\left.\frac{dL_{\alpha}}{dt}\right|_{T_{a}}$and$\left.\frac{dl_{\alpha}}{dt}\right|_{T_{0}}$and between $\left.\frac{dL_{\beta}}{dt}\right|_{T_{b}}$and $\left.\frac{dl_{\beta}}{dt}\right|_{T_{0}}$show that both the remelting and resolidification rate are much larger at the transverse direction (5 times), indicating that the liquid film migrates nearly laterally towards the center of the primary dendrite stem during isothermal annealing. It can be easily deduced from the analysis above that even though the density of classical heterogeneous nucleation sites like grain boundaries or free surface for melting is low in the directionally solidified samples, the reduction of initial superheating in the solid is fast enough to change the concentration to the local equilibrium values in the bulk within a few minutes. The two solid/liquid and liquid/solid interfaces can migrate at a high rate because the release and consumption of latent heat balance each other [3].

**3. Migration of primary/peritectic interface within secondary dendrite arm**

On the secondary dendrite arms, besides the migration of α/β interface due to migration of liquid film, the migration of secondary dendrite arms by the TGZM effect has been observed in several alloys including peritectic systems [4, 5]. During the migration of secondary dendrite arms, solidification/remelting occur at both the front and back edges of the secondary dendrite arms simultaneously. In peritectic systems, solidification of peritectic phases occurs at the front side of the secondary dendrite arms, and remelting of peritectic phase, then the primary phase enclosed by it occurs simultaneously at the back edge of the secondary dendrite arms. Therefore, the variation in morphology of the α/β interface is more complex than that in primary dendrite arms during isothermal annealing. And the migration of the α/β interface is influenced by all these factors such as migration of liquid film and migration of secondary dendrite arm itself. Depending on whether the liquid film exists at the α/β interface which is in the vicinity of the front/back edge of the secondary dendrite arm, the migration process is analyzed as follows. It should be noted that the liquid film can not migrate laterally in this case since the solute concentrations of these triple junctions are equal to each other in isothermal condition.

**3.1 Migration of liquid film near the front edge of the secondary dendrite arm**

Based on the discussion above, when liquid phase nucleates at the α/β interface near the front side of the secondary dendrite arms, both the concentration difference across the liquid film and the TGZM effect should be taken into consideration. The thickness of this liquid film along the temperature gradient direction is assumed to be *L_a_*, the temperatures of the front and back edges of the liquid film are denoted as T_a1_ and T_a2_, respectively. Considering the initial coarsening by referring to Fig. 2(a):

$T_{a1}=T_{\beta}+m_{l}^{\beta}C_{l}^{\beta,T_{a1}}$ (14a)

$T_{a2}=T_{\alpha}+m_{l}^{\alpha}C_{l}^{\alpha,T_{a2}}$ (14b)

where $C_{l}^{\beta,T_{a1}}$and $C_{l}^{\alpha,T_{a2}}$are the solute concentrations of liquid at T_a1_ and T_a2_, respectively. Simultaneously, due to assumptions 3 and 4, concentration gradient resulting from temperature gradient is established within the interdendritic liquid layer:

$T_{a1}=T_{a2}+GL_{a}$ (14c)

Combining the above three equations:

$C_{l}^{\beta,T_{a1}}-C_{l}^{\alpha,T_{a2}}=-\frac{1}{m_{l}^{\alpha}}\left[\left(m_{l}^{\alpha}-m_{l}^{\beta}\right)\left(C_{LP}-C_{l}^{\beta,T_{a1}}\right)-GL_{a}\right]$ (15)

The section $-\frac{1}{m_{l}^{\alpha}}\left[\left(m_{l}^{\alpha}-m_{l}^{\beta}\right)\left(C_{LP}-C_{l}^{\beta,T_{a1}}\right)\right]$ corresponds to the concentration difference across this liquid film due to local equilibrium in an isothermal condition. By relating to our previous work, the section $\frac{GL_{a}}{m_{l}^{\alpha}}$ corresponds to the TGZM effect [6]. It can be obtained that since $\left[\left(m_{l}^{\alpha}-m_{l}^{\beta}\right)\left(C_{LP}-C_{l}^{\beta,T_{a1}}\right)-GL_{a}\right]<0$, so $C_{l}^{\alpha,T_{a2}}>C_{l}^{\beta,T_{a1}}$, indicating that the concentration difference across this liquid film due to local equilibrium is not important in comparison with the TGZM effect. Here the requirement that $G>\frac{1}{L_{a}}\left[\left(m_{l}^{\alpha}-m_{l}^{\beta}\right)\left(C_{LP}-C_{l}^{\beta,T_{a1}}\right)\right]$ is surely satisfied since the right hand side of is $\left[\left(m_{l}^{\alpha}-m_{l}^{\beta}\right)\left(C_{LP}-C_{l}^{\beta,T_{a1}}\right)\right]$always negative.

According to the Fick’s First law:

$J_{1}=D\frac{C_{l}^{\alpha,T_{a2}}-C_{l}^{\beta,T_{a1}}}{L_{a}}$ (16)

The application of a mass balance for a small displacement *dL_a_* of the α/β interface results in the following differential equation:

$J_{2}=C_{l}^{\beta,T_{a1}}\left(k_{\beta}-1\right)\left.\frac{dL_{a}}{dt}\right|_{T_{a1}}$ (17)

$J_{1}=J_{2}$ (18)

$\left.\frac{dL_{a}}{dt}\right|_{T_{a1}}=\frac{D}{m_{l}^{\alpha}C_{l}^{\beta,T_{a1}}\left(k_{\beta}-1\right)L_{a}}\left[\left(m_{l}^{\alpha}-m_{l}^{\beta}\right)\left(C_{LP}-C_{l}^{\beta,T_{a1}}\right)-GL_{a}\right]$ (19a)

Thus, the remelting of peritectic β phase induced by the TGZM effect occurs at the front edge of the liquid film. Similarly, at the back edge of liquid film:

$\left.\frac{dL_{a}}{dt}\right|_{T_{a2}}=\frac{D}{m_{l}^{\alpha}C_{l}^{\alpha,T_{a2}}\left(1-k_{\alpha}\right)L_{a}}\left[\left(m_{l}^{\alpha}-m_{l}^{\beta}\right)\left(C_{LP}-C_{l}^{\beta,T_{a1}}\right)-GL_{a}\right]$ (19b)

The directions of these remelting/resolidification velocities are illustrated in Fig. 2(a1). In a word, the liquid film migrates upwards due to the TGZM effect. By referring to assumptions 2–3, the relation between $C_{l}^{\beta,T_{a1}}$and $C_{l}^{\alpha,T_{a2}}$is:

$C_{l}^{\alpha,T_{a2}}-C_{l}^{\beta,T_{a1}}=T_{P}\cdot\left(\frac{1}{m_{l}^{\alpha}}-\frac{1}{m_{l}^{\beta}}\right)-\frac{T_{a2}}{m_{l}^{\alpha}}+\frac{T_{a1}}{m_{l}^{\beta}}$ (20a)

$C_{l}^{\alpha,T_{a2}}-C_{l}^{\beta,T_{a1}}=\left(T_{P}-T_{a1}\right)\cdot\left(\frac{1}{m_{l}^{\alpha}}-\frac{1}{m_{l}^{\beta}}\right)+\frac{GL_{a}}{m_{l}^{\alpha}}$ (20b)

Comparison between Eqs. (19a) and (19b) shows that the remelting rate at T_a1_ is larger than the solidification rate at T_a2_, thus, the liquid film should be elongated along the temperature gradient direction.

**3.2 Migration of liquid film near the back edge of the secondary dendrite arm**

Considering the liquid film located at temperatures T_b1_ to T_b2_, referring to Fig. 2(b):

$T_{b1}=T_{\alpha}+m_{l}^{\alpha}C_{l}^{\alpha,T_{b1}}$ (21a)

$T_{b2}=T_{\beta}+m_{l}^{\beta}C_{l}^{\beta,T_{b2}}$ (21b)

where $C_{l}^{\alpha,T_{b1}}$and $C_{l}^{\beta,T_{b2}}$are the solute concentrations of liquid at T_b1_ and T_b2_, respectively. Simultaneously, due to assumptions 3 and 4, concentration gradient resulting from temperature gradient is established within the liquid film:

$T_{b1}=T_{b2}+GL_{b}$ (21c)

Combining the above three equations:

$C_{l}^{\beta,T_{b2}}-C_{l}^{\alpha,T_{b1}}=\frac{1}{m_{l}^{\beta}}\left[\left(m_{l}^{\beta}-m_{l}^{\alpha}\right)\left(C_{LP}-C_{l}^{\alpha,T_{b1}}\right)-GL_{b}\right]$ (22)

Similar to what we have discussed above, $\frac{1}{m_{l}^{\beta}}\left[\left(m_{l}^{\beta}-m_{l}^{\alpha}\right)\left(C_{LP}-C_{l}^{\alpha,T_{b1}}\right)\right]$ corresponds to the concentration difference across this liquid film due to local equilibrium in isothermal condition. And $-\frac{GL_{b}}{m_{l}^{\beta}}$ corresponds to the TGZM effect. Since$C_{l}^{\beta,T_{b2}}>C_{l}^{\alpha,T_{b1}}$, so$\left[\left(m_{l}^{\beta}-m_{l}^{\alpha}\right)\left(C_{LP}-C_{l}^{\alpha,T_{b1}}\right)-GL_{b}\right]<0$, and the concentration difference across this liquid film due to local equilibrium is not important in comparison with the TGZM effect. However, since these two parts are all positive, the TGZM effect accelerates the remelting/resolidification process of the liquid film located at the back edge of the secondary dendrite arm.

According to the Fick’s First law:

$J_{1}=D\frac{C_{l}^{\beta,T_{b2}}-C_{l}^{\alpha,T_{b1}}}{L_{b}}$ (23)

The application of a mass balance for a small displacement *dL_b_* of the α/β interface results in the following differential equation:

$J_{2}=C_{l}^{\alpha,T_{b1}}\left(k_{\alpha}-1\right)\left.\frac{dL_{b}}{dt}\right|_{T_{b1}}$ (24)

$J_{1}=J_{2}$ (25)

$\left.\frac{dL_{b}}{dt}\right|_{T_{b1}}=\frac{D}{m_{l}^{\beta}C_{l}^{\alpha,T_{b1}}\left(k_{\alpha}-1\right)L_{b}}\left[\left(m_{l}^{\beta}-m_{l}^{\alpha}\right)\left(C_{LP}-C_{l}^{\alpha,T_{b1}}\right)-GL_{b}\right]$ (26a)

Similarly, for the back edge of the liquid film:

$\left.\frac{dL_{b}}{dt}\right|_{T_{b2}}=\frac{D}{m_{l}^{\beta}C_{l}^{\beta,T_{b2}}\left(1-k_{\beta}\right)L_{b}}\left[\left(m_{l}^{\beta}-m_{l}^{\alpha}\right)\left(C_{LP}-C_{l}^{\alpha,T_{b1}}\right)-GL_{b}\right]$ (26b)

Similarly, the directions of these remelting/resolidification velocities are illustrated in Fig. 2(b1). By referring to assumptions 2–3, the relation between $C_{l}^{\alpha,T_{b1}}$and $C_{l}^{\beta,T_{b2}}$is:

$C_{l}^{\beta,T_{b2}}-C_{l}^{\alpha,T_{b1}}=\left(T_{P}-T_{b2}\right)\cdot\left(\frac{1}{m_{l}^{\alpha}}-\frac{1}{m_{l}^{\beta}}\right)-\frac{GL_{b}}{m_{l}^{\alpha}}$ (27)

The TGZM effect restricts the remelting/resolidification process of β phase induced by the concentration difference across the liquid film. Thus, the remelting of peritectic β phase induced by the TGZM effect occurs on the front edge of the liquid film. Comparison between Eq. (26a) and (26b) also shows that the remelting rate at T_b1_ is larger than the solidification rate at T_b2_, thus, the liquid film is also elongated along the temperature gradient direction. The parameters for calculation can be found in the previous work [7, 8].

**4. Interphase angles of the triple junction during migration process**

In the present work, the morphology of the liquid film located at the α/β interface is usually appears to be ellipse/ellipsoid in two/three dimensions. As shown in Fig. 3(a), if the morphology of this liquid phase is assumed to be circle/sphere in two/three dimensions, the interphase angles between primary α phase, peritectic β phase and liquid phase are illustrated. Furthermore, the directions of these three interfacial energies are also presented. In the case of a spherical morphology, if the interfacial equilibrium between these three phases is satisfied at the triple junction point P, then there exists:

$\frac{\gamma_{\alpha\beta}}{\sin\theta_{\alpha\beta}}=\frac{\gamma_{L\alpha}}{\sin\theta_{L\alpha}}=\frac{\gamma_{L\beta}}{\sin\theta_{L\beta}}$ (28)

It can be obtained from Fig. 3(a) that *θ_Lα_* and *θ_Lβ_* equal to π/2 and *θ_αβ_* is π/2, and Eq. (28) is not meaningful in this case. Thus, the morphology of the liquid phase should be identical to that shown in Fig. 3(b), which satisfies the requirement of interfacial equilibrium between these three phases. As the migration process proceeds, since the resolidification velocity at the tail of the liquid film is larger than the remelting velocity at the front of the liquid film, the morphology of the liquid film will be crescent, as that illustrated in Fig. 3(c). And both *θ_Lα_* and *θ_Lβ_* increase while *θ_αβ_* decrease, leading to variation in these three interfacial energies.

**References**

[1] J. D. Verhoeven, E. D. Gibson, interface stability of the melting solid-liquid interface: I. Sn-Sb alloys, J. Cryst. Growth, 1971; 11:29-38.

[2] M. Rettenmayr, Melting and remelting phenomena, Int. Mater. Rev. 54 (2009) 1-17.

[3] M. Buchmann, M. Rettenmayr, Microstructure evolution during melting and resolidification in a temperature gradient, J. Cryst. Growth, 2005; 284:544-553.

[4] H. Nassar, H. Fredriksson, On peritectic reactions and transformations in low-alloy steels, Metall. Mater. Trans. A, 2000; 41A:2776-2783.

[5] H. F. Lopez, Analysis of solute segregation effects on the peritectic transformation, Acta Metall. Mater. 1991; 39(7):1543-1548.

[6] H. Y. Lü, S. M. Li, L. Liu, H. Z. Fu, Peritectic phase growth in directionally solidified Cu-70%Sn alloy, Sci. China. G, 2007; 50(4):451-459.

[7] P. Peng, X.Z. Li, D.M. Liu, Y.Q. Su, J.J. Guo, H.Z. Fu, Effect of peritectic reaction on dendrite coarsening in directionally solidified Sn-36at.%Ni alloy, J. Mater. Sci. 47 (2012) 6108-6117.

[8] Y. Wu, T.J. Piccone, Y. Shiohara, M.C. Flemings, Dendritic growth of undercooled nickel-tin: Part II, Metall. Trans. A 18A (1987) 925-932.


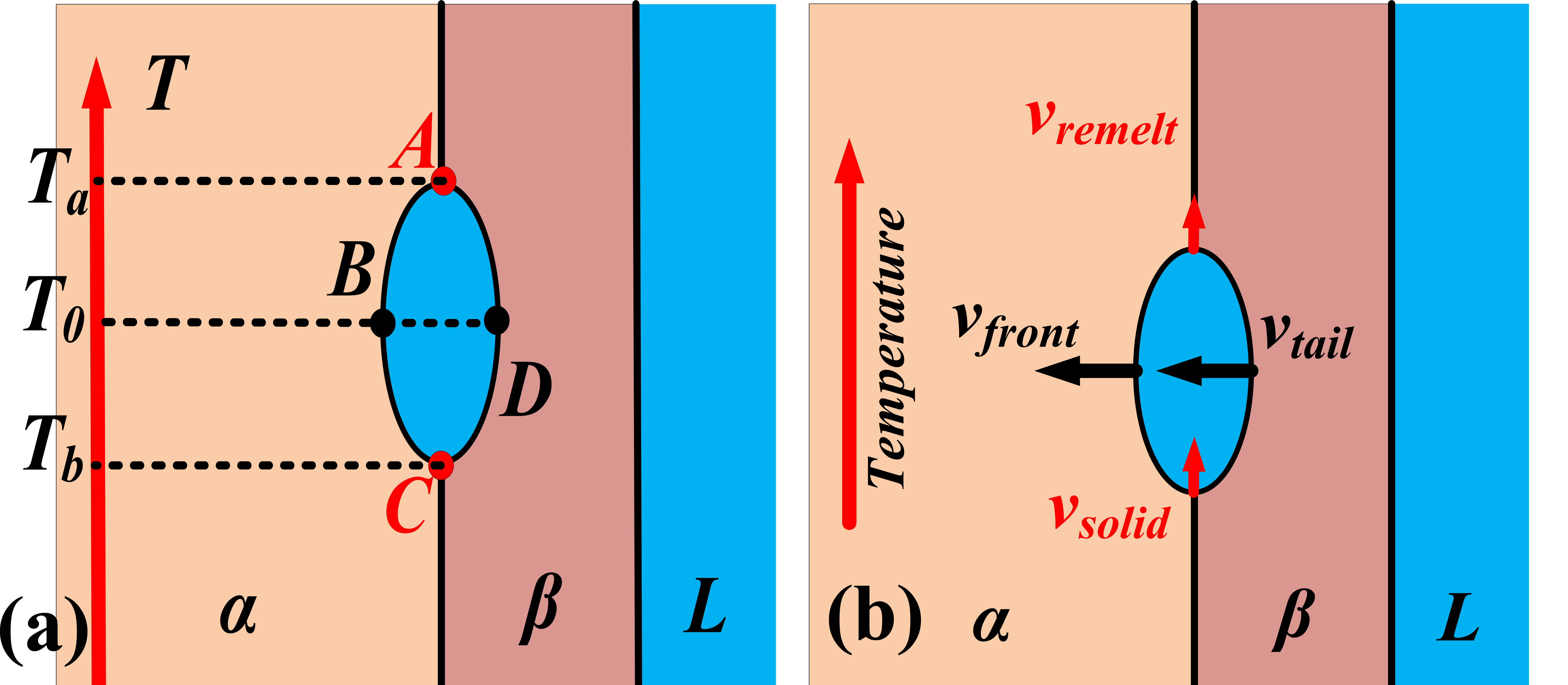


**Fig. 1.** Illustration of migration process of liquid film located at the primary dendrite arm: (a) dependence of temperatures of liquid film on positions of themselves and (b) different migration velocities at different positions of the liquid film.


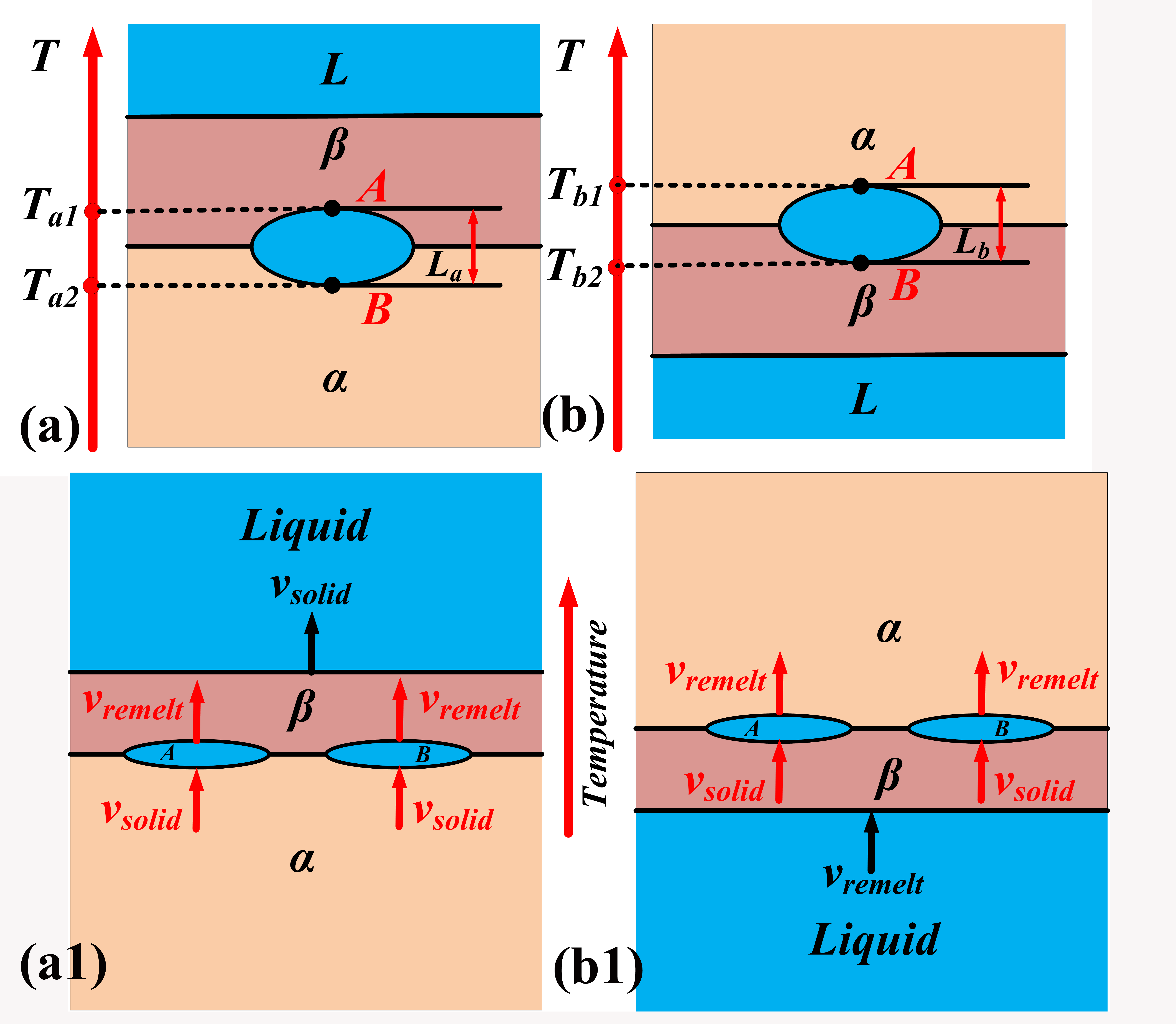


**Fig. 2.** Illustration of migration process of liquid film located at front ((a), (a1)) and back ((b), (b1)) edges of the secondary dendrite arm: dependence of temperatures of liquid film on positions of themselves ((a), (b)) and (b) different migration velocities at different positions of the liquid film ((a1), (b1)).


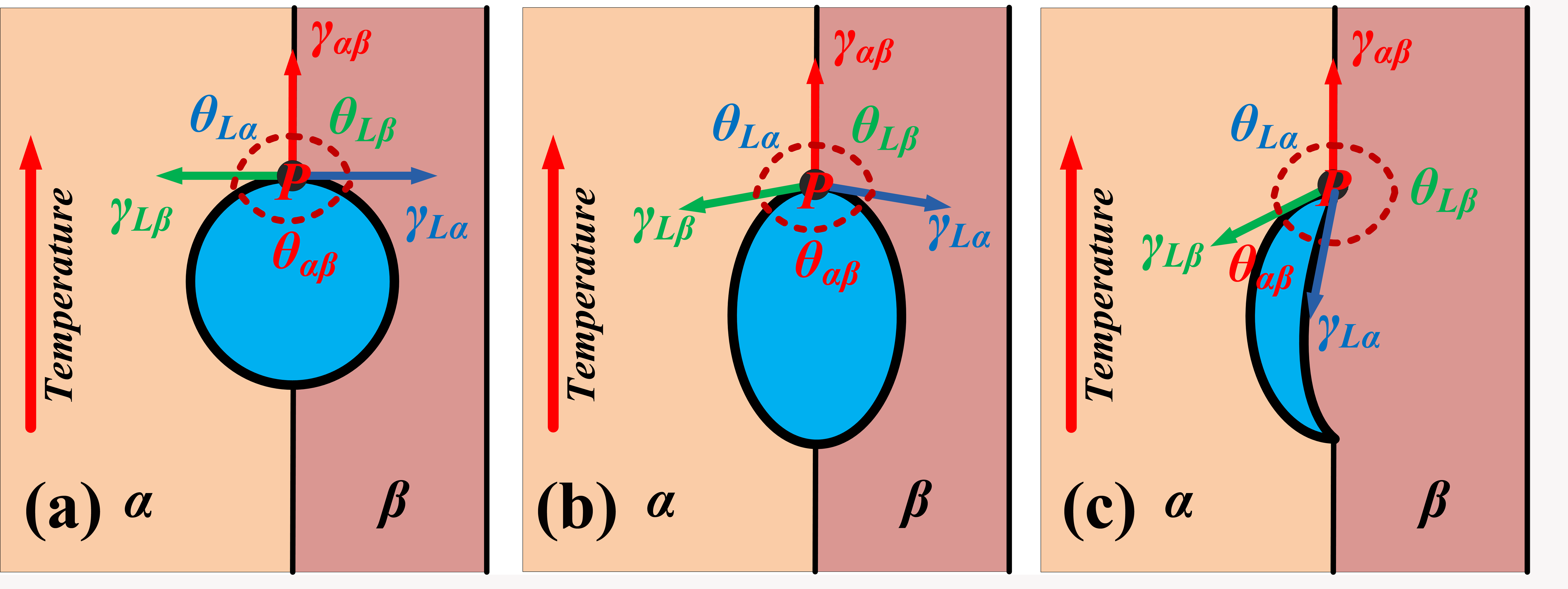


**Fig. 3.** Illustration of interphase angles and directions of interfacical energies when the morphology of the liquid phase located at the α/β interface is different: (a) the liquid phase is assumed to be spherical, (b) the liquid phase is assumed to be ellipsoid, which is the real morphology of the liquid phase, (c) the liquid phase is assumed to be crescent as migration proceeds.

1. ^^ Corresponding author at: School of Physical Science and Technology, Lanzhou University, Lanzhou 730000, PR China.

   Tel: +86 931 2166588.

   *E-mail address*: [pengp@lzu.edu.cn](mailto:lzupplzu@163.com) [↑](#footnote-ref-2)
